# Infectivity, transmission and pathogenicity of H5 highly pathogenic avian influenza clade 2.3.4.4 (H5N8 and H5N2) United States index viruses in Pekin ducks and Chinese geese

**DOI:** 10.1186/s13567-017-0435-4

**Published:** 2017-06-07

**Authors:** Mary J. Pantin-Jackwood, Mar Costa-Hurtado, Kateri Bertran, Eric DeJesus, Diane Smith, David E. Swayne

**Affiliations:** Exotic and Emerging Avian Viral Diseases Research Unit, Southeast Poultry Research Laboratory, U.S. National Poultry Research Center, Agricultural Research Service, U.S. Department of Agriculture, 934 College Station Rd, Athens, GA 30605 USA

## Abstract

In late 2014, a H5N8 highly pathogenic avian influenza (HPAI) virus, clade 2.3.4.4, spread by migratory waterfowl into North America reassorting with low pathogenicity AI viruses to produce a H5N2 HPAI virus. Since domestic waterfowl are common backyard poultry frequently in contact with wild waterfowl, the infectivity, transmissibility, and pathogenicity of the United States H5 HPAI index viruses (H5N8 and H5N2) was investigated in domestic ducks and geese. Ducks infected with the viruses had an increase in body temperature but no or mild clinical signs. Infected geese did not show increase in body temperature and most only had mild clinical signs; however, some geese presented severe neurological signs. Ducks became infected and transmitted the viruses to contacts when inoculated with high virus doses [(10^4^ and 10^6^ 50% embryo infective dose (EID_50_)], but not with a lower dose (10^2^ EID_50_). Geese inoculated with the H5N8 virus became infected regardless of the virus dose given, and transmitted the virus to direct contacts. Only geese inoculated with the higher doses of the H5N2 and their contacts became infected, indicating differences in infectivity between the two viruses and the two waterfowl species. Geese shed higher titers of virus and for a longer period of time than ducks. In conclusion, the H5 HPAI viruses can infect domestic waterfowl and easily transmit to contact birds, with geese being more susceptible to infection and disease than ducks. The disease is mostly asymptomatic, but infected birds shed virus for several days representing a risk to other poultry species.

## Introduction

The Asian-origin H5N1 A/goose/Guangdong/1/1996 (Gs/GD) lineage of highly pathogenic avian influenza (HPAI) viruses has spread across several continents affecting wild birds, poultry and humans. Despite great efforts to control H5N1 HPAI viruses, these viruses continue to circulate and evolve, which has led to the emergence of multiple genotypes or sublineages and the generation of reassortant H5 strains with novel gene constellations. Subclade 2.3.4.4 H5N1 viruses have mixed with several neuraminidase subtypes to generate widely circulating H5N2, H5N5, H5N6, and H5N8 subtypes of H5 HPAI viruses [1–6]. In early 2014, outbreaks of H5N8 HPAI virus were reported in South Korea and Japan in poultry and wild aquatic birds, with migratory aquatic birds strongly suspected in playing a key role in the spread of the virus [7, 8]. In late autumn 2014, H5N8 HPAI viruses were detected in Siberia, several countries in Europe, in South Korea, and in Japan [3, 4]. Concurrently, this virus was detected in the United States (U.S.) in captive falcons, wild birds, and backyard aquatic and gallinaceous poultry [5]. In addition, another novel reassortant H5 HPAI clade 2.3.4.4 virus (H5N2) was identified as the cause of an outbreak in poultry farms in British Columbia, Canada, during November 2014 [9], and was subsequently detected in the U.S. in wild waterfowl, raptors, and backyard poultry, including domestic ducks and geese [10]. From March to June 2015, this H5N2 virus predominated in the U.S., with extensive inter-farm transmission occurring in the Midwestern region. Over 7.5 million turkeys and 42.1 million chickens died or were depopulated in the USA during this outbreak which ended in June 2015 [11].

Wild and domestic waterfowl have played an important role in the maintenance and spread of Gs/GD lineage H5 HPAI viruses. Infected migratory waterfowl contributed to the spread of H5N1 and H5N8 HPAI viruses from Asia to other parts of the world [4, 8, 12, 13]. In 2016, H5N8 viruses of the same Gs/GD H5 lineage (HA clade 2.3.4.4) have been again detected in wild waterfowl in Russia, Europe, Middle East and Africa, these viruses causing outbreaks in poultry in many countries [14–16]. Domestic waterfowl have an important role in the maintenance and spread of H5N1 HPAI viruses, and have been shown to serve as intermediaries in the transmission of these viruses between wild waterfowl and other poultry species [17–19]. Wild waterfowl and domestic ducks are also important in the emergence and maintenance of H5N8 HPAI [3]. Hill et al. [20] found that wild waterfowl migration and domestic duck density were important factors in the epidemiology of H5N8 in the Republic of Korea. During the U.S. outbreak, H5N8 and H5N2 HPAI viruses were detected in backyard poultry including waterfowl [21], with possible contact with wild waterfowl or ponds reported in some cases [22]. H5N8 HPAI viruses have also affected commercial duck facilities in Europe and in the U.S. [16, 23, 24]. Recently, non-Gs/GD lineage H5 HPAI viruses (H5N1, H5N2, H5N9) have also caused outbreaks in domestic ducks in France in [25].

H5N1 Gs/GD lineage HPAI viruses are highly lethal to chickens; however, in domestic ducks these viruses can produce a range of clinical outcomes from asymptomatic infections to severe disease with mortality [26]. Both sick and asymptomatic infected ducks can shed high virus quantities into the environment favoring increased risk of transmission and potential outbreaks in commercial poultry. Naturally or experimentally, mortality in ducks caused by HPAI viruses had been infrequently reported before the Gs/GD H5N1 HPAI outbreaks in Asia [27, 28]. However, many Gs/GD lineage H5N1 HPAI viruses, and recently other Gs/GD-derived H5 viruses, have caused disease and death in domestic ducks (reviewed in [26]) [15, 16, 23, 24, 29]. Similarly, in domestic geese, outcome of infection with H5N1 HPAI viruses depends on the virus strain and the geese species. Domestic geese, naturally or experimentally infected with H5N1 viruses, showed from no clinical signs to neurological signs with or without mortality [30–35]. H5N8 and H5N2 HPAI viruses have also been detected in wild geese [16, 36]. In Taiwan, H5 clade 2.3.4.4 viruses (H5N2, H5N3 and H5N8) produced a severe epidemic in the domestic geese population in 2015, and more than 2.2 million geese died or were culled [37]. Recently, infections with H5N8 HPAI viruses have also been reported in domestic geese in Europe [16].

The recent emergence and recurrence of outbreaks of H5NX Gs/GD lineage HPAI in poultry underscore the need to better understand the pathobiology of these viruses in domestic waterfowl. In this study, in order to improve early detection of H5 HPAI viruses in domestic waterfowl, the infectivity, transmissibility and pathogenicity of the index H5N8 and H5N2 clade 2.3.4.4 HPAI viruses from the U.S. outbreak, was investigated in Pekin ducks and Chinese geese.

## Materials and methods

### Virus

The highly pathogenic avian influenza (HPAI) viruses A/gyrfalcon/Washington/40188-6/2014 H5N8 (GF/WA/14 H5N8) and A/Northern Pintail/Washington/40964/2014 H5N2 (NP/WA/14 H5N2) were used as challenge viruses. These were the first two Gs/GD H5 HPAI isolates, HA clade 2.3.4.4, from the U.S. outbreak and are considered representative of both the wholly Eurasian H5N8 lineage viruses and reassortant Eurasian/North American lineage H5N2 viruses, respectively [5]. The viruses were propagated and titrated in specific pathogen free (SPF) embryonating chicken eggs (ECE) using standard methods [38]. Stocks were diluted to the target dose with brain–heart infusion (BHI) broth (Becton, Dickinson and Company, Sparks, MD, USA). The experiments were performed in biosecurity level-3 enhanced (BSL-3E) facilities in accordance with procedures approved by the U.S. National Poultry Research Center (USNPRC) Institutional Biosecurity Committee.

### Animals and housing

Pekin ducks (*Anas platyrhynchos var. domestica*) and White Chinese geese (*Anser cygnoides*) were obtained at 2 days of age from a commercial hatchery and reared in USNPRC facilities. At 2 weeks of age, birds were transferred to ABSL-3 enhanced facilities for virus challenge. Serum samples were collected from ten birds from each species prior to challenge to ensure that the birds were serologically negative for AI viruses by ELISA (FlockCheck Avian Influenza MultiS-Screen Antibody Test^®^, IDEXX Laboratories, Westbrook, ME, USA). Each experimental group was housed separately in self-contained isolation units ventilated under negative pressure with inlet and exhaust HEPA-filtered air within the animal BSL-3 enhanced facilities at Southeast Poultry Research Laboratory. This study and associated procedures were reviewed and approved by the USNPRC Institutional Animal Care and Use Committee (IACUC).

### Experimental design and sampling

Similar experiments were conducted with each bird species to evaluate the mean bird infectious dose (BID_50_), transmissibility, and pathogenicity of the 2014 H5 HPAI index viruses. The 2-week-old ducks and geese were separated into virus-inoculated groups (4–5 birds for each species) as shown in Table 1. Non-inoculated control groups were included for each species. Groups containing 4–5 Pekin ducks were challenged with the appropriate dose per bird (10^2^ 50% egg infectious doses [EID_50_] per bird [low dose], 10^4^ EID_50_ per bird [medium dose], or 10^6^ EID_50_ per bird [high dose]), administered in 0.1 mL by the intrachoanal route. To examine pathogenesis, two additional birds were challenged with the high dose of the viruses. Sham-inoculated control ducks were inoculated with 0.1 mL of sterile allantoic fluid diluted 1:300 in BHI. To assess transmission by contact, three naïve ducks were introduced in the isolators with virus-inoculated ducks at one day post-inoculation (dpi). Groups containing four Chinese geese were challenged by intrachoanal inoculation with sham inoculum or with a dose of 10^2^, 10^4^, or 10^6^ EID_50_/bird in 0.1 mL of either virus (Table 1). Two additional geese were challenged with the high dose of the viruses. Two naïve geese were introduced in the isolators with virus-inoculated geese at 1 dpi. The inoculum titers were subsequently verified by back titration in ECE as 1.7–1.9 (low dose), 3.5–3.9 (medium dose), and 5.7–6.1 (high dose) log_10_ EID_50_/0.1 mL.

**Table 1:** **Mortality, number of birds infected, 50% bird infectious doses, and seroconversion of 2-week-old Pekin ducks and Chinese geese inoculated by the intrachoanal route and contact-exposed to A/gyrfalcon/WA/40188-6/2014 (H5N8) and A/Northern pintail/WA/40964/2014 (H5N2) HPAI viruses**

Species	Virus	Inoculated birds					Contact-exposed birds		
		Virus dose (log_10_ EID_50_)	# of infected birds/total^a^	BID_50_^b^ (log_10_ EID_50_)	Mortality^c^ (dpi^d^)	Serology^d^ (range of antibody titers, log_2_)	# of infected birds/total	Mortality (dpe^e^)	Serology (range of antibody titers, log_2_)
Pekin ducks	H5N8	2	0/5	3.0	0/5	0/5	0/3	0/3	0/3
		4	5/5		0/5	5/5 (4–5)	1/3	0/3	1/3 (4)
		6	5/5		0/5	5/5 (4–5)	2/3	0/3	2/3 (4)
	H5N2	2	0/5	3.0	0/5	0/5	0/3	0/3	0/3
		4	5/5		0/5	5/5 (3)	2/3	0/3	1/3 (3)
		6	4/4		0/4	4/4 (3–4)	3/3	0/3	3/3 (3–5)
Chinese geese	H5N8	2	4/4	< 2	0/4	1/4 (4)	2/2	0/2	0/2
		4	4/4		0/4	0/4	2/2	0/2	1/2 (3)
		6	4/4		0/4	2/4 (3)	2/2	1/2 (11)	0/2
	H5N2	2	0/4	3.0	0/4	0/4	0/2	0/2	0/4
		4	4/4		0/4	1/4 (3)	2/2	1/2 (10)	0/1
		6	4/4		1/4 (8)	1/3 (3)	2/2	0/2	0/2

^a^Number of birds infected/total number of birds inoculated; determined by qRRT-PCR and serology.

^b^BID_50_, mean bird infectious dose.

^c^Number of dead birds/total number of birds inoculated (days post-inoculation).

^d^Number of birds with positive antibody titers/total number of birds inoculated.

^e^Days post-exposure.

Clinical signs were monitored daily for 10 days in the duck experiment and for 11 days in the geese experiment. Oropharyngeal (OP) and cloacal (CL) swabs were collected from all birds at days 2, 4, 7 and 10, 11 to determine virus shed. Body temperatures were taken at 2 and 4 dpi from birds inoculated with the high dose of the viruses and from the sham inoculated control birds. Significant difference for body temperatures between groups was analyzed using Kruskal–Wallis test (GraphPad Prism™ Version 5 software). A *p* value of < 0.05 was considered to be significant. For each species, the two additional birds challenged with the high dose of the viruses were euthanized and necropsied at 4 dpi to evaluate gross lesions. A full set of tissues were collected from each bird and fixed in 10% neutral buffered formalin solution, paraffin-embedded, sectioned, and stained with hematoxylin-and-eosin for histopathologic evaluation. Duplicate sections were stained by immunohistochemical (IHC) methods to determine influenza viral antigen distribution in individual tissues [39]. Portions of lung, heart, brain, muscle and spleen were also collected and stored at −80 °C for subsequent virus detection and quantification. Sera were collected from all surviving birds at the end of the experiments to evaluate infection status by antibody levels using hemagglutination inhibition (HI) assay. HI assays were performed using standard methods and homologous antigen [40]. The virus infectious dose was calculated by the Reed–Muench method [41], using the criteria that birds were considered infected if they shed detectable levels of virus at any time and/or were positive for antibody at the end of the study.

### Viral RNA quantification in swabs and tissues

OP and CL swabs were collected in 1 mL of BHI broth with a final concentration of 10 μg/mL of gentamicin, 100 units/mL of penicillin G, and 56 μg/mL of amphotericin B, and kept frozen at −80 °C until processed. RNA was extracted using MagMAX™-96 AI/ND Viral RNA Isolation Kit^®^ (Ambion, Inc.) following the manufacturer’s instructions. qRRT-PCR reactions targeting the influenza virus M gene [42] were conducted using AgPath-ID one-step RT-PCR Kit (Ambion, Austin, TX, USA) and the ABI 7500 Fast Real-Time PCR system (Applied Biosystem, Carlsbad, CA, USA). Viral RNA was extracted from tissues using Trizol LS reagent (Invitrogen, Carlsbad, CA, USA) and the Qiagen RNeasy Mini Kit (Qiagen Corp, Valencia, CA, USA). In tissue homogenates, and in order to standardize the amount of nonspecific RNA from the tissue, the resulting viral RNA extracts were quantified by NanoDrop™ 1000 Spectrophotometer (Thermo Fisher Scientific) following the manufacturer’s instructions and accordingly diluted with phosphate buffered saline to obtain 50 ng/µL. For virus quantification, a standard curve was established with RNA extracted from dilutions of the same titrated stock of the challenge virus. Results were reported as EID_50_/mL or EID_50_/g equivalents and the lower limit of detection was was 10^1.8^ EID_50_/mL for both viruses. For statistical purposes, qRRT-PCR negative samples were given a numeric value of 1.7 log_10_ EID_50_/mL (1.7 log_10_ EID_50_/g).

## Results

### Infectivity, transmission and pathogenicity of the H5N8 and H5N2 HPAI viruses in domestic ducks

No ducks were infected in the groups inoculated with the lowest dose (10^2^ EID_50_) of each virus (Table 1). Birds were considered infected if they had detectable virus or seroconverted. All ducks inoculated with the medium (10^4^ EID_50_) or high (10^6^ EID_50_) dose of the viruses were infected but no mortality occurred. The 50% bird infectious dose (BID_50_) for both viruses was 3 log_10_ EID_50_. One or 2 of 3 contact ducks in the groups inoculated with the medium dose for both viruses were infected, and 2 or 3 of 3 contact ducks in the high dose groups were infected. No mortality was observed in these ducks either.

**Figure 1:**
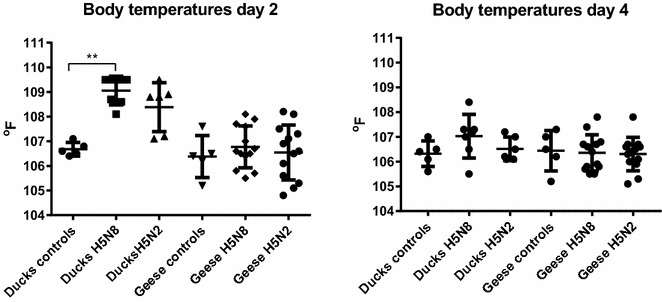
**Mean body temperatures of 2-week-old Pekin ducks and Chinese geese inoculated by the intrachoanal route with 10** ^**6**^ **EID**_**50**_ **of H5N8 and H5N2 HPAI viruses, at 2 and 4 days post-inoculation.** Bars represent the standard deviation of the mean. Significant difference in body temperature compared to controls (**p < 0.01).

A significant difference in body temperature was observed at 2 dpi, but not at 4 dpi, between sham-inoculated control ducks and H5N8-inoculated ducks (high dose group) (Figure 1). Four of six ducks inoculated with the H5N2 virus also showed high body temperatures. Conjunctivitis, tearing of the eyes, and diarrhea was observed intermittently in a low percentage of ducks in each group (1–2 ducks). One duck in the H5N2 high dose group presented mild ataxia starting at 2 dpi. At the end of the study (10 dpi) one duck from the H5N8 medium dose group and a second duck from the H5N2 high dose group also had mild ataxia. No other clinical signs were observed in the virus-inoculated and contact ducks. At 4 dpi, two ducks inoculated with the high dose of the viruses were euthanized for gross examination. One of the ducks inoculated with the H5N8 virus had airsacculitis and marbled spleen. No gross lesions were observed in the three other ducks.

### Infectivity, transmission and pathogenicity of the H5N8 and H5N2 HPAI viruses in domestic geese

All geese inoculated with the H5N8 virus were infected regardless of the dose given (BID_50_ was less than 2 log_10_ EID_50_) (Table 1). No geese were infected in the group inoculated with the lowest dose of the H5N2 virus, but the virus did infect all geese at the medium and high doses (BID_50_ was 3 log_10_ EID_50_). No differences in body temperature were found among virus-inoculated geese (high dose groups) at 2 and 4 dpi when compared to sham-inoculated controls (Figure 1). In the groups of geese inoculated with the H5N8 virus, no or mild clinical signs (conjunctivitis) were observed during the study, but at 11 dpi one goose from the high dose group had mild tremors and incoordination, and one of the contacts from the same group was found dead. In the groups of geese inoculated with the H5N2 virus, one goose and a contact goose from the high and medium dose groups presented severe ataxia and torticollis, at 8 and 10 days respectively, and were euthanized and necropsied. At 11 dpi, one goose from each of the medium and high dose groups had mild ataxia. The rest of the geese inoculated with H5N2 showed no or mild clinical signs (conjunctivitis). No gross lesions were observed in the two geese that were euthanized and examined at 4 dpi from the H5N8 pathogenesis group. The two geese from the H5N2 pathogenesis group had multifocal areas of necrosis in the pancreas. The goose from the H5N2 high dose group euthanized at 8 dpi due to severe neurological signs had nasal discharge and empty intestines. The contact goose from the H5N2 medium dose group euthanized at 10 dpi had a pale spleen, enlarged heart, hemorrhagic thymus, malacic brain, and nasal discharge.

### Microscopic lesions and viral antigen distribution

**Figure 2:**
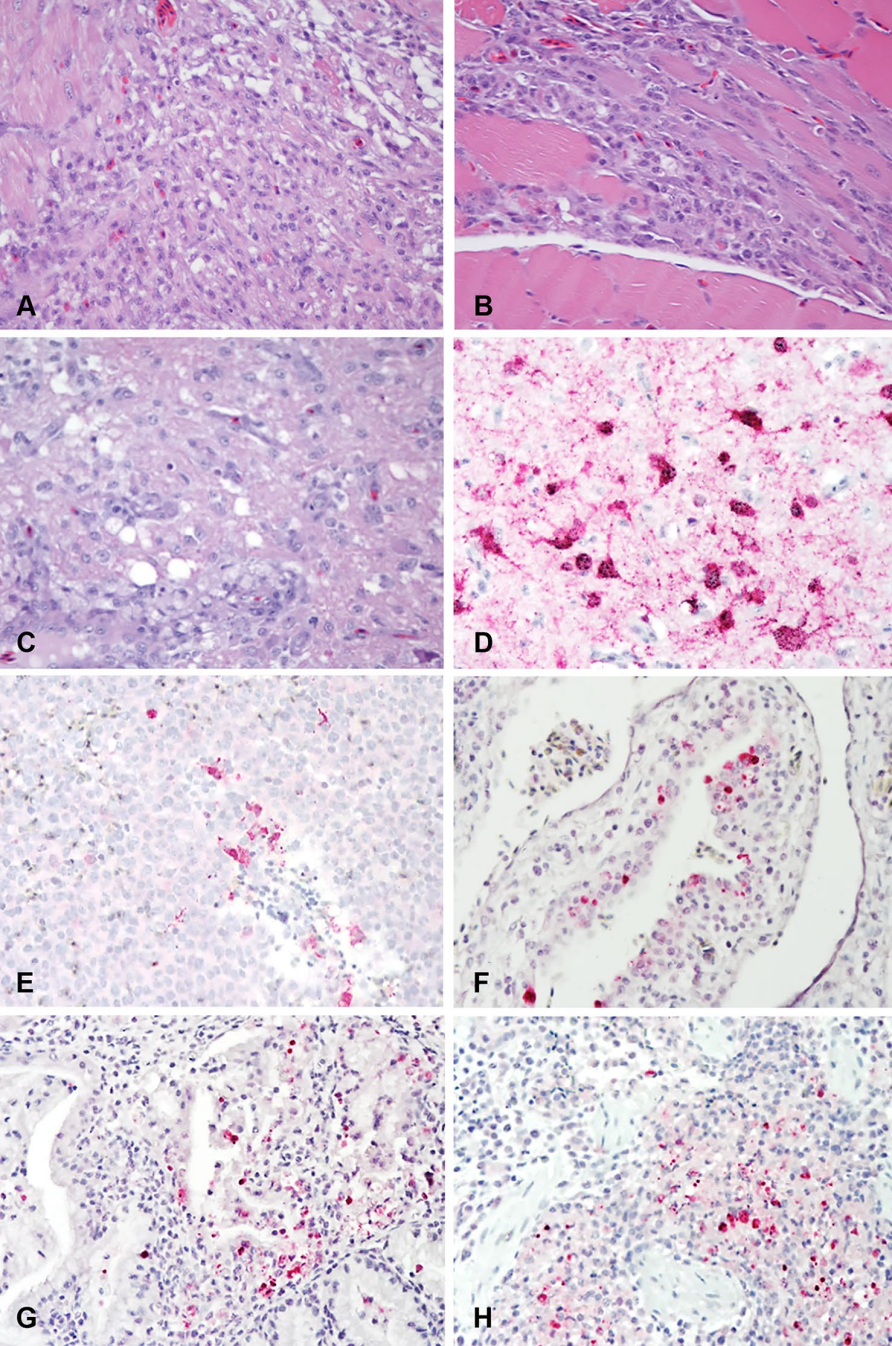
**Histological lesions and immunohistochemical detection of viral antigen in 2-week old Pekin ducks and Chinese geese inoculated by the intrachoanal route with H5N8 and H5N2 HPAI viruses. 40×; viral antigen staining in red.** **A** Heart. Goose inoculated with H5N2, 8 dpi; lymphoplasmacytic inflammation. **B** Skeletal muscle. Goose inoculated with H5N2, 10 dpi; lymphoplasmacytic inflammation. **C** Cerebrum. Goose inoculated with H5N2, 8 dpi; foci of malacia, and gliosis. **D** Cerebrum. Goose inoculated with H5N2, 10 dpi; viral antigen in neurons and glial cells. **E** Spleen. Goose inoculated with H5N2, 8 dpi; viral antigen in necrotic cells and mononuclear cells. **F** Airsac. Pekin duck inoculated with H5N8, 4 dpi; viral antigen present in epithelial cells. **G** Harderian gland. Pekin duck inoculated with H5N8, 4 dpi; viral antigen present in epithelial cells and infiltrating monocytes. **H** Lung. Pekin duck inoculated with H5N8, 4 dpi; viral antigen in epithelium of air capillaries and infiltrating monocytes.

In order to evaluate microscopic lesions and sites of virus replication, tissues collected from ducks and geese necropsied at 4 dpi were examined, and immunohistochemical staining for AI virus nucleoprotein antigen was performed (Table 2; Figure 2). Tissues from the two geese that were euthanized at 8 and 10 dpi were also examined.

**Table 2:** **AI virus antigen immunohistochemical staining in tissues from Pekin ducks and Chinese geese inoculated by the intrachoanal route or contact-exposed to H5N8 and H5N2 HPAI viruses**

Species	Virus	Detection of AI virus antigen in tissues													
		Nasal ep.	Trachea	Lung	Air sacs	Brain	Heart	Spleen	Liver	Skeletal muscle	Pancreas	Cloacal bursa	Cecal tonsils	Harderian gland	Thymus
Pekin ducks 4 dpi	H5N8	++/+	+/++	+/++	+++/+	+/+	+/+	++/+	−/−	+/+	−/−	+/−	−/−	++/++	−/−
	H5N2	+/+	+/+	+/−	+/+	−/+	−/−	−/−	−/−	+/−	−/−	+/−	−/−	−/−	+/−
Chinese geese 4 dpi	H5N8	+/++	+/−	++/+	+/−	+/+	+/++	++/+	−/−	+/−	−/−	−/−	−/−	−/+++	−/+
	H5N2	++/++	+/+	++/++	+/na	++/++	++/+++	++/++	+/+	+/−	+/+	+/++	++/++	−/−	++/+
Chinese geese 8 and 10 dpi	H5N2	+/+	+/+	++/++	na/na	+++/+++	++/++	++/+	+/+	+/+	+/+	+/++	+/+	na/na	+/−

Birds were euthanized at 4, 8 or 10 dpi (bird 1/bird 2).

Kidney, adrenal glands, and intestine were negative for virus antigen staining.

− no positive cells, + single positive cells, ++ scattered groups of positive cells, +++ widespread positivity, *na* not available.

In all ducks and geese examined at 4 dpi, mild catarrhal rhinitis and sinusitis was observed. The tracheas had mild degenerative changes of the overlying epithelium and mild lymphocytic infiltration in the submucosa. Also present: mild to moderate interstitial pneumonia, mild airsacculitis, focal necrosis of the epithelia of the Harderian glands, mild proliferation of gut-associated lymphoid tissues, and mild to moderate lymphoid depletion in cloacal bursa, thymus and spleen. Remaining organs lacked significant histopathologic changes. The two geese from the H5N2 pathogenesis group had, in addition, multifocal areas of necrosis in the pancreas, and the two geese examined at 8 and 10 dpi had individual cell necrosis of myofibers and focal mononuclear inflammation in the heart and thigh skeletal muscle (Figures 2A and B), and in the brain, randomly scattered foci of malacia, gliosis and perivascular cuffing (Figure 2C). Viral antigen staining was present in multiple tissues from ducks and geese infected with the H5 HPAI viruses indicating systemic infection (results in Table 2; Figures 2C–G). Viral antigen was present in epithelial cells and infiltrating phagocytes in the nasal turbinates, trachea, bronchus, lung air capillaries, air sac, Harderian glands, cloacal bursa, and in resident and infiltrating phagocytes of the spleen. Viral antigen staining was also present in pancreatic acinar epithelial cells, hepatocytes, neurons and glial cells of the brain, fragmented cardiac and skeletal myofibers of geese infected with the H5N2 virus.

### Replication of H5N8 and H5N2 HPAI viruses in Pekin ducks and Chinese geese

**Figure 3:**
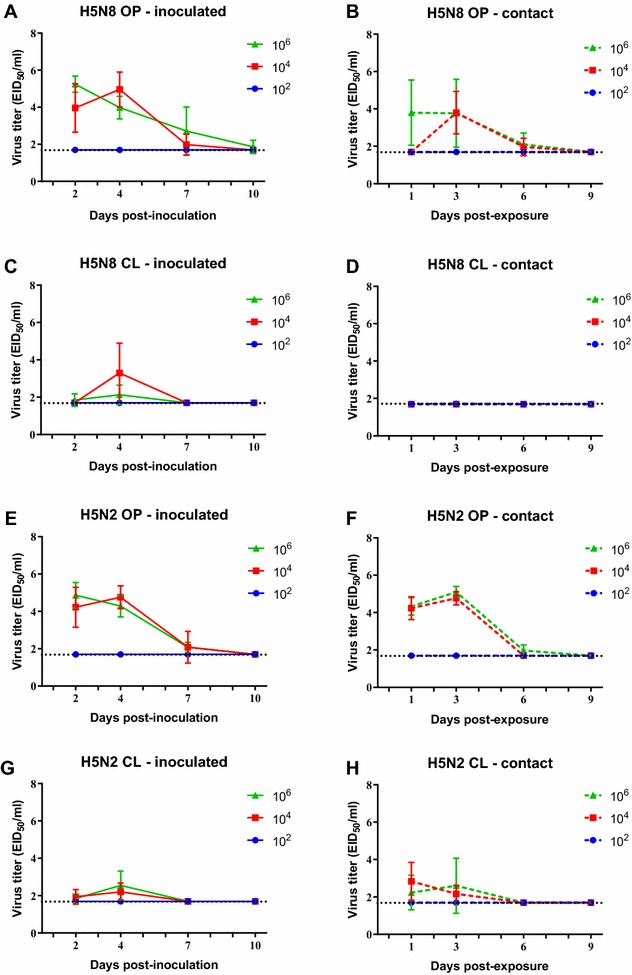
**Mean oropharyngeal (OP) and cloacal (CL) viral shed from 2-week-old Pekin ducks directly inoculated (A, C, E, G) or contact-exposed (B, D, F, H) with H5N8 and H5N2 HPAI viruses**. Ducks were inoculated with 10^2^, 10^4^ and 10^6^ EID_50_ of either virus and titers determined by qRRT-PCR. Bars represent the standard deviation of the mean. Swabs from which virus was not detected were given a numeric value of 10^1.7^ EID_50_/mL.

**Figure 4:**
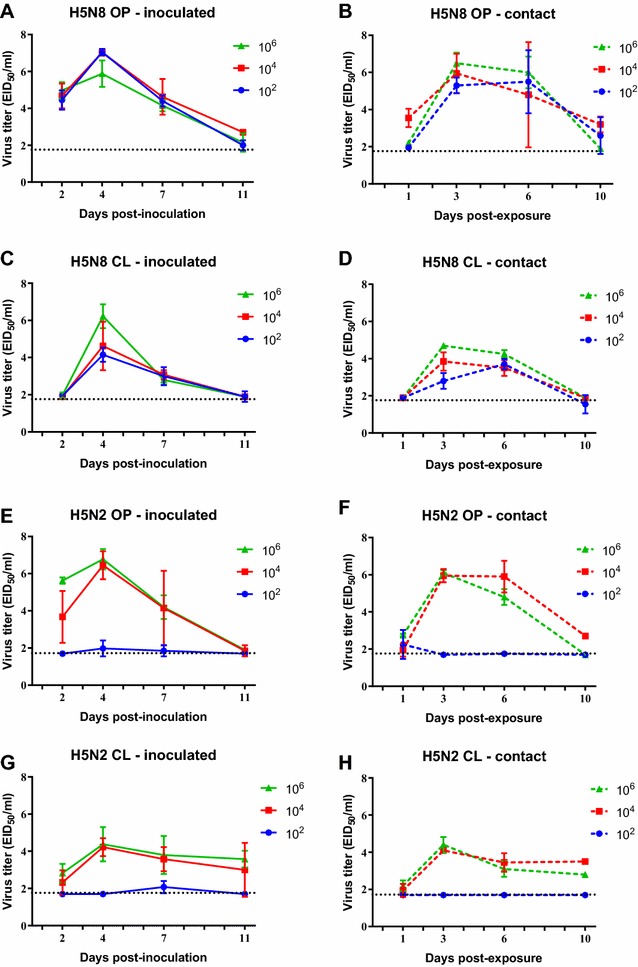
**Mean oropharyngeal (OP) and cloacal (CL) viral shed from 2-week-old Chinese geese directly inoculated (A, C, E, G) or contact-exposed (B, D, F, H) with H5N8 and H5N2 HPAI viruses**. Geese were inoculated with 10^2^, 10^4^ and 10^6^ EID_50_ of either virus and titers determined by qRRT-PCR. Bars represent the standard deviation of the mean. Swabs from which virus was not detected were given a numeric value of 10^1.7^ EID_50_/mL.

Quantitation of viral shed was performed by qRRT-PCR using extrapolation of a standard curve generated with the challenge viruses via virus isolation and titration. OP and CL viral shed was examined at 2, 4, 7 and 10 dpi in ducks (Figures 3A, C, E and G) and at 2, 4, 7 and 11 dpi in geese (Figures 4A, C, E and G). To evaluate viral shed in contact birds, naïve birds were introduced in the isolator with inoculated birds at 1 dpi and sampled at 1, 3, 6 and 9 or 10 days post-exposure (dpe) (Figures 3 and 4B, D, F and H).

Ducks inoculated with the low dose of either the H5N8 or H5N2 virus did not shed virus and virus was not transmitted to contacts. Ducks inoculated or contact-exposed in the medium and high dose groups shed virus mostly through the OP route (Figures 3A, B, E and F); few ducks (including the contacts) shed through the CL route and, if so, at very low titers (Figures 3C, D, G and H). Ducks from the medium and high dose groups shed virus by the OP route up to 7 dpi (6 dpe for contacts). Ducks inoculated with the high doses of the viruses were able to transmit to 3–4 of the 6 contacts, but higher titers were shed by the H5N2 contacts (Figures 3F and H).

All geese inoculated with H5N8 virus shed virus through the OP and CL routes at high titers regardless of the inoculation dose (Figures 4A and C). H5N8 and H5N2 contact exposed geese became infected and showed a similar virus shedding pattern as the directly inoculated geese (Figures 4B and D). Only geese inoculated with the medium and high dose of the H5N2 virus shed virus (Figure 2E, G). Contact geese in these groups also shed virus similar to the inoculated birds (Figures 4F and H). H5N2 OP viral titers were similar to those observed with the H5N8 virus, but H5N2 virus was shed for longer by the CL route. Overall, the Pekin ducks shed lower virus titers and for a shorter period of time (7 days vs 11 days) than the Chinese geese. The peak of viral shedding for both viruses in both species was between 3 and 5 dpi.

**Figure 5:**
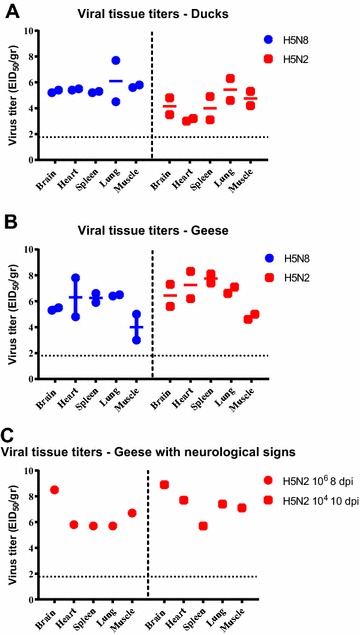
**Virus titers in tissues from Pekin ducks and Chinese geese directly inoculated or contact-exposed with H5N8 and H5N2 HPAI viruses.** Titers determined by qRRT-PCR. **A** Tissues from Pekin ducks, 4 dpi. **B** Tissues from geese, 4 dpi. **C** Tissues from geese showing neurological signs euthanized at 8 dpi and 10 dpi inoculated or exposed to H5N2 virus, respectively. Bars represent the standard error of the mean. Tissues from which virus was not detected were given a numeric value of 10^1.7^ EID_50_/g.

To evaluate systemic replication of H5N8 and H5N2 HPAI viruses in ducks and geese, viral titers were determined in brain, heart, spleen, lung and muscle. Tissues were collected from 2 birds of each 10^6^ EID_50_ inoculated groups at 4 dpi (Figure 5). Ducks and geese showed moderate to high H5N8 and H5N2 HPAI virus titers in all tissues examined, with the highest titers found in tissues of geese inoculated with the H5N2 virus. Tissues were also collected from the two geese exposed to H5N2 virus and showing neurological signs; both birds also presenting high virus titers in all tissues, especially in brain.

### Serology

Serum samples were examined for detectable titers of antibodies against the corresponding challenge virus at 10 or 11 dpi, for Pekin ducks or geese respectively. All Pekin ducks inoculated with the medium and high dose of either virus seroconverted (Table 1); 1 of 3 contacts placed in the medium dose groups and 2 or 3 of 3 in the highest dose groups seroconverted. Only one goose inoculated with the low dose, two geese inoculated with the high dose and one contact goose in the medium dose group seroconverted to the H5N8 virus. Two geese, one from the medium dose and one from the high dose group seroconverted to the H5N2 virus.

## Discussion

In this study we describe the pathogenesis and transmission dynamics of the U.S. index H5N8 and H5N2 HPAI viruses (Gs/GD lineage, HA clade 2.3.4.4) in domestic ducks and geese with the objective of better understanding the infection process in order to improve strategies for early detection of HPAI viruses in domestic waterfowl. Based on viral shed and serology results, only ducks inoculated with the medium and high doses of the viruses (10^4^ and 10^6^ EID_50_, respectively) were infected and were able to efficiently transmit to contacts, resulting in a BID_50_ of 3 log_10_ EID_50_ for both viruses. Similarly, geese inoculated with the medium and high doses of the H5N2 HPAI virus and the contact birds in these groups were infected, resulting also in a BID_50_ of 3 log_10_ EID_50_. In contrast to ducks, all geese inoculated with the low, medium and high doses of the H5N8 HPAI virus were infected and transmitted the virus to contacts, with a resulting BID_50_ of < 2 log_10_ EID_50_. These findings support the conclusion that geese were more susceptible than ducks to infection by the H5N8 virus.

Although most ducks and geese in this study survived virus infection and showed only minimal clinical signs, occasionally neurological signs were present in both species but were more common and severe in geese. Virus was detected by qRRT-PCR and IHC in the brain of both ducks and geese, but virus titers were higher and presence of viral antigen was more common in the brain of infected geese, especially in geese infected with the H5N2 virus. In general, virus replication in tissues was higher in the geese than in the ducks. This higher virus replication and more severe clinical signs in geese compared to ducks has been also observed in other studies comparing H5N1 HPAI virus infections side by side using these species [31, 32]. In addition, geese shed both viruses for longer times and in higher titers, especially by the CL route, than ducks, indicating that the viruses tested in this study replicated better in this species.

The Gs/GD H5N1 HPAI viruses developed the unique capacity among HPAI viruses to infect and cause disease in domestic waterfowl and wild birds producing a range of syndromes from asymptomatic infections to systemic disease and death [31]. The pathogenicity of Gs/GD lineage H5N1 HPAI viruses in waterfowl is associated with the efficiency of virus replication [43]. Viral dissemination to the brain, leading to severe neurological dysfunction, is considered one of the causes of the high virulence of H5N1 viruses in ducks, but lesions to other important organs could lead to multi-organ failure and death [44–47]. In addition to the virus strain, the susceptibility of wild and domestic waterfowl to H5N1 HPAI virus infection and the presentation of disease vary depending on other factors, including the age and species of the birds and management practices [19, 48]. In wild geese, neurological disease along with systemic virus replication and mortality was observed following experimental inoculation with Gs/GD lineage H5N1 HPAI viruses [49, 50]. Canada geese (*Branta canadiensis*) experienced sporadic deaths in natural H5N1 HPAI virus outbreaks [51–54], and have shown high susceptibility to the virus as evidenced by systemic replication and high mortality rates after experimental infection [34, 50, 55], although age-related differences in susceptibility were described [56]. Similar findings have been reported for other species of wild geese, including bar-headed geese (*Anser indicus*) and cackling geese (*B. utchinsii*) [49, 57]. Domestic geese naturally infected with H5N1 HPAI virus showed severe clinical signs including neurological signs [33]. Domestic geese (*Anser anser var. domestica*) experimentally infected with H5N1 viruses presented neurological signs and 40–50% mortality [34]. Similarly, domestic White Chinese geese (*Anser cygnoides*) showed high mortality when experimentally infected [30]. However, Embden geese (*Anser anser var. domestica*) and Graylag geese (*Anser anser*) inoculated with different H5N1 HPAI viruses developed neurological signs, but lacked mortality [31, 32]. These observations indicate that both the virus strain and waterfowl species affect the outcome of infection with Gs/GD lineage H5 viruses, similar to what was observed in our study.

Most Pekin ducks infected with either of the H5 viruses examined in this study had high body temperatures at 2 dpi when compared to controls, but this difference was only significant in the H5N8 group. An increase in body temperature after infection with the same H5N8 virus was seen in our previous studies with mallards [58, 59], and it has also been reported with H5N1 virus infections in several breeds of *Anas platyrrhynchs var. domestica* ducks (Pekin, Mallards, Black Runners, Rouen, Khaki Campbell), but not in Muscovy ducks (*Cairina moschata*) [60, 61]. Similar to Muscovy ducks, no increase in body temperature was observed in the geese, indicating differences in the innate immune response between Pekin ducks and geese, which could also explain the differences in disease severity. Most surviving geese examined at 11 dpi did not have antibodies against the challenge virus, contrary to most ducks, also indicating differences in humoral immune responses between these two species. This low seroconversion rate in geese after AI virus infection was also reported in our previous study examining the pathogenesis of a H7N9 virus in different avian species [62]. The immunological differences observed between waterfowl species could affect virus infection, replication, tissue tropism, and virus clearing, consequently affecting the severity of the pathogenic outcome observed.

When comparing the present study with our previous studies examining the pathobiology of the same H5N8 and H5N2 HPAI viruses in mallards, we observe that both viruses are more infectious for mallards (BID_50_ of < 2 log_10_ EID_50_) than for Pekin ducks (3 log_10_ EID_50_) [58, 59]. Similar to Pekin ducks, infected mallards showed minimal clinical signs and transmitted the viruses to all contacts; however, mallards shed virus for longer than Pekin ducks. Interestingly, when examining two H5N2 HPAI viruses isolated later in the U.S. outbreak from commercial poultry, both had a similar high infectivity as the index H5N2 virus in mallards, but one of the viruses showed lower replication and one caused some mortality when given at high doses [58]. These results show that individual H5N2 Gs/GD clade 2.3.4.4 viruses have different pathobiology in infected ducks, and that the duck type can also affect the pathogenic outcome of infection.

Likewise, a range of pathobiological outcomes, from no clinical signs to severe disease including neurological signs, were observed in wild and domestic ducks experimentally inoculated with H5N8 viruses of the same Gs/GD lineage (clade 2.3.4.4), with mortality rates varying from 0 to 20% [1, 29, 58, 59, 63–68], and the viruses transmitting efficiently to naïve contacts [63–65, 67, 68]. A study describing the pathobiologic characteristics of a H5N1 virus isolated in Canada also belonging to HA clade 2.3.4.4, found that the virus was highly pathogenic to juvenile Muscovy ducks and adult Chinese geese causing systemic infections with neurological signs and mortality (36 and 22% respectively) in both species [69]. The virus was also efficiently transmitted and caused mortality (40 and 80% respectively) in naïve contact ducks and geese of the same species.

Natural infections of domestic ducks with H5N8 HPAI viruses have been associated with disease and mortality, but such mortality is typically low. Hungary reported a H5N8 HPAI outbreak during late winter of 2015 at a Pekin duck fattening facility [70]. In addition to increased mortality in the flock and respiratory symptoms, the affected birds showed lethargy and neurological signs. The H5N8 HPAI outbreak in Korea in 2014 mostly resulted in drops in egg production [63], and the outbreak in the United Kingdom in 2014 showed a gradual reduction in egg production and mild increased mortality over a 7-day period [24]. Similarly, an outbreak with H5N8 HPAI virus in commercial Pekin ducks in California in early 2015 resulted in decreased feed consumption, moderate increase in mortality and severe neurological signs in 2% of the ducks [23]. In these natural infections other factors could have increased the severity of disease presentation. In line with this, fungal and bacterial lesions probably exacerbated the mortality and clinical presentation in the outbreak in the UK, motivating disease investigation [24]. If concurrent infections with other pathogens or added stress under intensive conditions do not prompt an avian notifiable disease investigation, the virus can continue to circulate with mild clinical signs or asymptomatically in domestic waterfowl and potentially spread to other holdings, resulting in a more extensive outbreak of HPAI.

As the Gs/GD H5 HPAI viruses continue to evolve and reassort, antigenic and genetic divergent strains have emerged, many expressing distinct pathobiological features and increased virulence for waterfowl. New H5N8 reassortants recently detected in Europe have caused disease and death in both domestic and wild ducks [15, 16], these viruses appearing to be more pathogenic for waterfowl and other wild bird species than the 2014–2015 H5N8 HPAI viruses.

In conclusion, the results of the present study indicate that infection of naïve domestic Pekin ducks and Chinese geese with clade 2.3.4.4 H5 HPAI viruses resulted in efficient virus replication and transmission to contacts. Mortality was low and clinical signs were uncommon, consisting mostly of mild neurological signs. In the field, clinical signs could be exacerbated by other infectious and non-infectious factors. Our findings emphasize the need to implement and improve active surveillance in domestic waterfowl (backyard and commercial), and increase biosecurity compliance to reduce direct and indirect contact between poultry and wild waterfowl in order to detect, prevent and control AI in poultry.

## Abbreviations

AI: avian influenza
Gs/GD: A/goose/Guangdong/1/1996
dpi: day post-inoculation
dpe: days post-exposure
ECE: embryonating chicken eggs
HA: hemagglutinin
HI: hemagglutination inhibition
HPAI: highly pathogenic avian influenza
LPAI: low pathogenic avian influenza
BID_50_: mean bird infectious dose
MDT: mean death time
EID_50_: mean egg infectious dose
na: not applicable
qRRT-PCR: quantitative real-time RT-PCR
SPF: specific pathogen free
